# Phenotypic variation of life‐history traits in native, invasive, and landrace populations of *Brassica tournefortii*

**DOI:** 10.1002/ece3.5747

**Published:** 2019-11-18

**Authors:** Brian Alfaro, Diane L. Marshall

**Affiliations:** ^1^ Department of Biology University of New Mexico Albuquerque New Mexico

**Keywords:** biological invasions, crop evolution, rapid evolution, stability, wild crop relatives

## Abstract

Varying environments can result in different patterns of adaptive phenotypes. By performing a common greenhouse experiment, we identified phenotypic differentiation on phenology, leaf morphology, branch architecture, size, and reproduction, among native, invasive, and landrace ranges of *Brassica tournefortii*. We first compared trait means and fitness functions among ranges, then we analyzed how trait means and selection strength of populations respond to varying aridity. Most traits varied such that landrace > invasive > native. Excluding reproduction, which was positively selected, most trait PCs experienced nonlinear selection in the native range but frequently shifted to directional selection in invasive and/or landrace ranges. The absence of strong clines for trait means in landrace and invasive populations suggest that agricultural practices and novel environments in source locations affected adaptive potential. Selection strength on faster reproductive phenology (negative directional) and leaf margin trait (disruptive) PCs coincided with increasing moisture. In native populations, higher aridity was associated with more days to reproduction, but landrace and invasive populations show stable mean time to reproduction with increasing moisture. A stable adaptive trait can increase range expansion in the invasive range, but stability can be beneficial for future harvest of *B. tournefortii* seed crops in the face of climate change.

## Graphical abstract

Varying environments can result in different patterns of adaptive phenotypes. By performing a common greenhouse experiment, we identified phenotypic differentiation in phenology, leaf morphology, branch architecture, size, and reproduction, among native, invasive, and landrace ranges of *Brassica tournefortii* (Sahara mustard). Understanding these differences may allow us to assess how native, agricultural, or invasive populations can be conserved, cultivated, or managed.

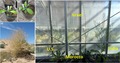

## 1 INTRODUCTION

Contrasting evolutionary scenarios among discrete groups of plant populations can produce diverse patterns of phenotypic differentiation. Depending on how (micro)evolutionary and ecological factors interact, local adaptation or phenotypic plasticity can alter correlations between trait values and environmental gradients or trait values and fitness (e.g., Conner & Hartl, 2004). When populations of the same species have experienced different histories and environments, we can examine evolution under a variety of selection pressures. For example, evolution of native plant populations can span geological timescales, while adaptations in crops and weeds are shaped by human activity (Meyer & Purugganan, 2013). Some varieties have been bred since the rise of civilizations, over a few thousand years (Purugganan & Fuller, 2009), while invasive populations can evolve rapidly in the span of a few decades or centuries because of the rapid changes in selective pressures in new environments (Bossdorf et al., 2005; Buswell, Moles, & Hartley, 2011; Colautti & Barrett, 2013; Dlugosch & Parker, 2008a, 2008b). Comparing populations of a single species that have evolved under these differing conditions allows us to assess effects of these mechanisms of selection on adaptive trait variation and association of candidate traits with environmental variation.

Because conditions in native, invasive and/or cultivated ranges of a species can vary, we may find different adaptations and associations of traits with environments among these types of populations. Moreover, anthropogenic factors, such as artificial selection and unintentional dispersal, can also affect patterns of phenotypic variation. Although traditional landraces are subjected to artificial selection for success under cultivation, these populations may still have ample evolutionary potential and therefore may show unique responses to environmental variation (Brush, 1995; Mercer, Martínez‐Vásquez, & Perales, 2008; Mercer & Perales, 2010). A different evolutionary scenario shapes phenotypic variation in invasive populations. First, human‐mediated dispersal of propagules can introduce individuals with limited genetic diversity to a new area. Then, genetic diversity of pioneer populations can increase or show structuring depending on the amount of gene flow from other introduced populations (Bartlett, Novak, & Mack, 2002; Dlugosch & Parker, 2008a, 2008b; Valliant, Mack, & Novak, 2007; Williams & Fishman, 2014). There may be introduced genotypes pre‐adapted to original conditions, but if the new habitat is different than the native range (e.g., discrete latitudinal ranges), then environmental filtering can structure traits differently via local adaptation (Bossdorf et al., 2005; Dlugosch & Hays, 2008; Dlugosch & Parker, 2008a, 2008b; Maron, Vila, Bommarco, Elmendorf, & Beardsley, 2004). Plasticity can also result in phenotypic clines across environmental gradients among invasive populations (Colautti & Lau, 2015; Matesanz, Horgan‐Kobelski, & Sultan, 2012), but this is not always the case (Godoy, Valladares, & Castro‐Diez, 2011; Matzek, 2012). Whether clines formed by invasive or crop populations will be the same or different than those of native populations will depend on associations of traits with the new environments and how these interactions shape evolution of phenotypes (Colautti, Maron, & Barrett, 2009).

Pairwise comparisons of invasive, native, and landrace populations have revealed important patterns of phenotypic evolution. For example, similar mean trait values and parallel/continuous clinal responses of invasive and native populations are considered signals that pre‐adapted genotypes established in similar habitat conditions in non‐native ranges (Bossdorf et al., 2005; van Kleunen, Schlaepfer, Glaettli, & Fischer, 2011). In contrast, differing means among populations or among ranges and intersecting trait‐environment clines indicate genotype‐by‐environment interaction and/or local adaptation to new environments (Colautti & Barrett, 2013; Colautti & Lau, 2015; Colautti et al., 2009). On one hand, analysis of clinal responses can tell us about evolution of invasive species; on the other hand, comparisons of phenotypic and genetic variation in wild and landrace populations allow us to examine the effects of domestication on plant evolution. While pairwise comparisons are informative, a three‐way examination of adaptive phenotypic response to environmental factors in native, invasive, and landrace ranges would provide additional insight because it can reveal evolutionary trends of plants potentially subjected to different types of selection. We are aware of no studies that explicitly compare phenotypic means, fitness functions, and clinal patterns of traits and selection strength along environmental gradients among native, invasive, and landrace ranges of a single species.

While testing genetic basis of traits is critical, determining fitness consequences confirms adaptive trait evolution (Conner & Hartl, 2004). But, merely describing fitness functions does not detect possible selection agents and how selection can change across landscapes. To determine possible environmental drivers of selection, some have regressed population mean trait values with associated environmental gradients (Colautti & Barrett, 2010; Maron et al., 2004). These putative selection agents can then be confirmed by regression of environmental variables versus selection gradients (Conner & Hartl, 2004; Stewart & Schoen, 1987; Wade & Kalisz, 1989, 1990).

To test how variation and selection of phenotypes can be restructured by different histories and climatic gradients, we chose a study system that has both landrace and invasive populations outside of an extant native range. Specifically, we used *Brassica tournefortii* (Sahara mustard) to test whether traits and their fitness, climate variables and traits, or climate variables and selection gradients, have similar or different relationships in native, invasive, and landrace ranges. To assess how adaptive trait variation and strength of selection can vary among ranges and among climatic gradients, we asked questions about phenotypic evolution in *B. tournefortii*:

1. Do the suites of phenology, leaf morphology, branch architecture, size, and reproductive traits vary among ranges, among population nested within ranges, and among maternal families nested within populations within ranges?
2. Which traits have significant fitness functions, and do these fitness functions vary among the native, invasive, and landrace ranges?
3. Do composite trait means (mean factor scores) vary along climate gradients to form clines, and do these potential clines vary among native, invasive, and landrace ranges?
4. Do composite traits vary in strength of selection among populations along climate gradients, and do regression lines of environmental variation versus selection strength differ among native, invasive, and landrace ranges?

## 2 MATERIAL AND METHODS

### 2.1 Study species

**Figure 1:**
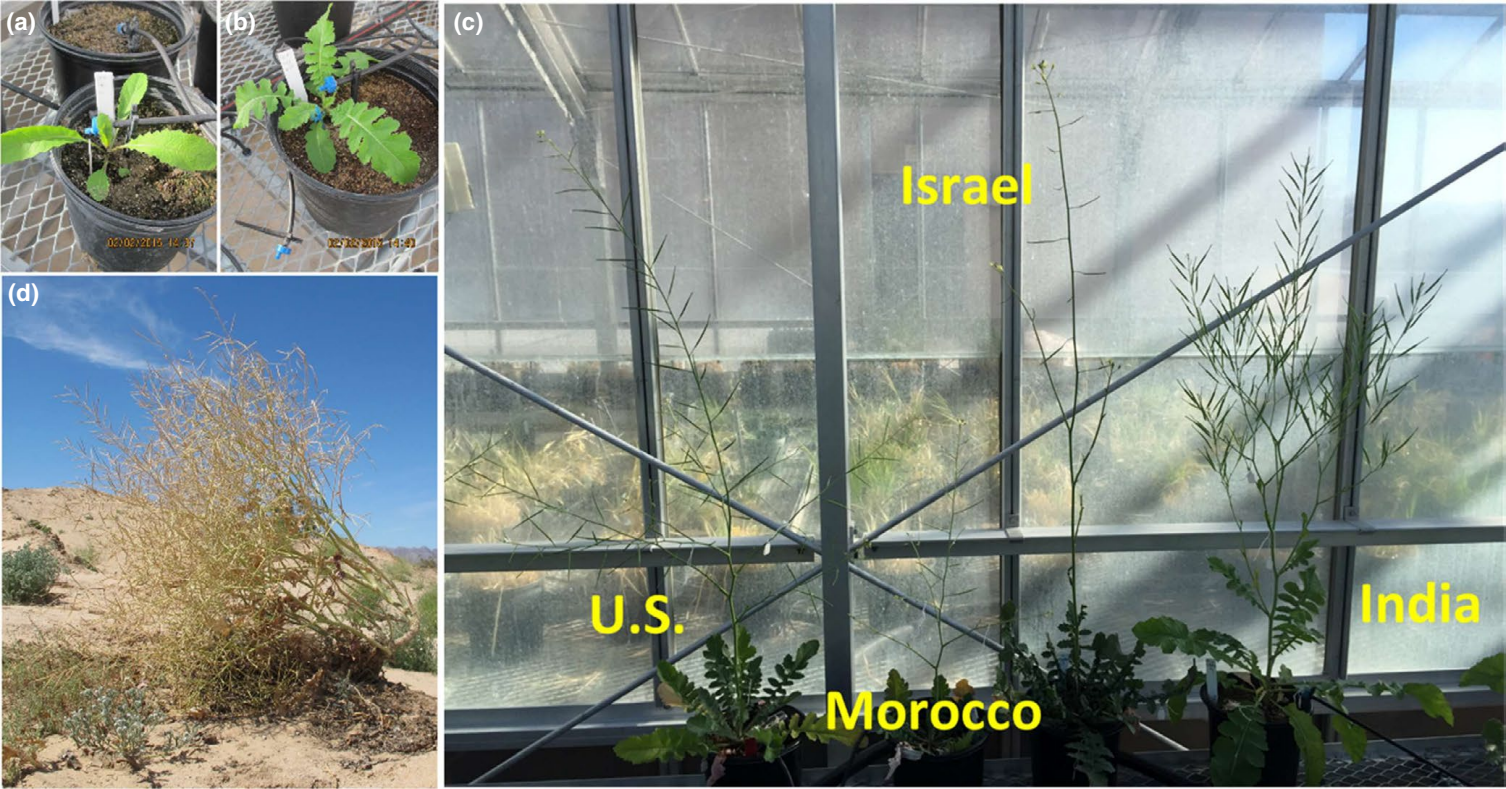
*Brassica tournefortii* seedlings/rosettes used as parental generation (a and b), showing variability in leaf margin morphology, (c) bolting/mature seeding plants from common greenhouse study, and (d) mature/senesced plant sampled for population genetic study in Mojave Desert, CA

*Brassica tournefortii* (Sahara mustard) is a xerophytic, self‐pollinating annual endemic to North Africa, the Middle East, and Mediterranean regions of Europe, is a seed crop in Pakistan and India, and is invasive in Australia and North America (Abella, Suazo, Norman, & Newton, 2013; Berry, Gowan, Miller, & Brooks, 2014; Boutsalis, Karotam, & Powles, 1999; Dimmitt, 2009; Gorecki, Long, Flematti, & Stevens, 2012). In the western United States, *B. tournefortii* is an invasive plant that outcompetes native desert flora and impacts small animals (Hulton VanTassel et al., 2014). In Australia, it is cataloged as a noxious agricultural weed (Gorecki et al., 2012). It was introduced to the western United States in the late 1920s and has spread in the last four decades; the invasive populations are therefore quite young. Thus, *B. tournefortii* has a wide global range and populations with diverse histories, making it ideal for examining plant phenotypic evolution. In the invasive ranges, it outcompetes endemic plants by having early and rapid phenology (Marushia, Brooks, & Holt, 2012; Marushia, Cadotte, & Holt, 2010), high fecundity (Bangle, Walker, & Powell, 2008; Trader, Brooks, & Draper, 2006), variable germination (Abd El‐Gawad, 2014; Bangle et al., 2008; Chauhan, Gill, & Preston, 2006; Gorecki et al., 2012), and natural and artificial dispersal modes that allow long‐distance migration (Berry et al., 2014; Li, Dlugosch, & Enquist, 2015). Based on our own pilot studies conducted in the greenhouse, different source populations can express variable morphological phenotypes and phenology (Figure 1a–c). In its invasive range in the deserts of the southwestern United States, a mature plant can grow as an entire diaspore that disperses seeds as a tumbleweed (Figure 1d).

### 2.2 Study area

**Figure 2:**
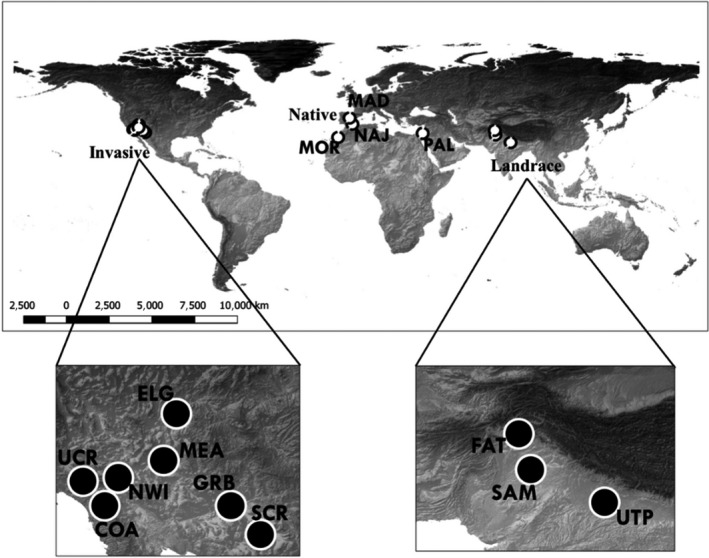
*Brassica tournefortii* sources used for experimental crosses. Invasive range: COA—east Coachella Valley (CA), NWI—North Indian Canyon Rd. (CA), UCR—University of California, Riverside (CA), SCR—Santa Cruz River (AZ), GRB—Gila River Basin (AZ), ELG—Elgin Rd. (NV), MEA—Lake Mead (NV). Native range: MOR—Tiznit, Morocco, MAD—Madrid, Spain, NAJ—Almeria, Spain, PAL—Palmachim, Israel. Landrace range: SAM—Sammundri, Pakistan, FAT—Fateh Jang, Pakistan, UTP—Uttar Pradesh, India

Our study included populations from native, invasive, and agricultural ranges of *B. tournefortii* (Figure 2, Table 1). For the native range, we used four populations from Morocco, Spain, and Israel. For the agricultural range, we used three populations from India and Pakistan that we call landraces because the seeds were collected from crops grown and bred via traditional practices and not through intensive commercial methods. The seeds we used to grow experimental populations for the native and landrace populations were obtained from accessions provided by the U.S. Department of Agriculture—Agricultural Research Services (USDA‐ARS) National Genetic Resources Program. For the invasive range, we used seven populations from the southwestern United States. The seeds from these populations were collected in 2008 by professional biologists who volunteered to sample in the southwestern United States. For each site, approximately 10 fruits per plant were collected from 10 to 12 plants per population; these fruits were collected separately for each maternal plant and stored in labeled coin envelopes.

**Table 1:** Source population locations and climatic conditions

Locality	Range	Latitude	Longitude	Altitude (m)	Total annual precipitation (mm)	Mean annual temperature (°C)	Aridity index
Coachella Valley East (COA)	Invasive	33.65	−116.66	1,352	508	13	0.24
Elgin Road, NV (ELG)	Invasive	36.73	−114.43	620	437	14	0.15
Lake Mead, NV (MEA)	Invasive	35.20	−114.57	200	161	19	0.05
North Indian Canyon Rd. (NWI)	Invasive	34.00	−116.57	528	212	19	0.08
Santa Cruz River (SCR)	Invasive	32.40	−111.14	628	316	21	0.12
U.C. Riverside (UCR)	Invasive	33.98	−117.30	491	371	17	0.17
Fateh Jang, Pakistan (FAT)	Landrace	33.57	72.60	507	635	22	0.36
Sammundri, Pakistan (SAM)	Landrace	31.06	72.94	174	367	25	0.18
Uttar Pradesh, India (UTP)	Landrace	26.85	80.91	124	1,011	26	0.5
Almeria, Spain (NAJ)	Native	36.96	−2.20	440	139	19	0.08
Madrid, Spain (MAD)	Native	40.40	−3.68	602	98	22	0.06
Palmachim, Israel (PAL)	Native	31.93	34.70	21	209	18	0.11
Tiznit, Morocco (MOR)	Native	29.71	−9.71	211	279	17	0.14

### 2.3 Generation of seed families

**Figure 3:**
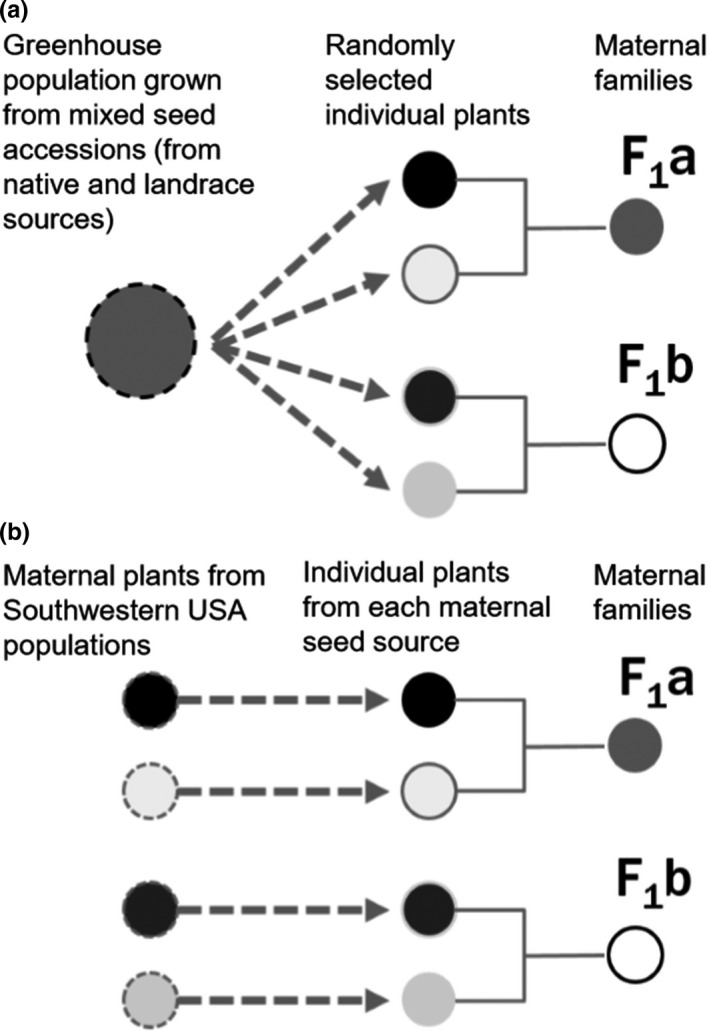
Diagrams of four hypothetical full‐sib families to illustrate the types of crosses used to generate seed families for native (a), landrace (a), and invasive (b) populations of *B. tournefortii*

To reduce maternal environmental effects and to avoid using plants with unknown parentage, we grew a parental generation in a common environment at the UNM Research Greenhouse for native, invasive, and landrace populations (Figure 3). In March of 2015, the resulting P_1_ plants were artificially crossed and their progeny were used for the experiment. For native and landrace populations, we created P_1_ generations using seed accessions from the USDA‐ARS. Each seed accession originated from field collection of seeds from about 15 to 30 plants per site, which were then maintained by the USDA in plant cages (Laura Marek, USDA, personal communication). We haphazardly drew seeds from each accession envelope, germinated, and grew 20 seeds per accession, and then crossed randomly paired individuals assigned as either maternal plants or pollen donors. The resulting F_1_ seed families from each artificial cross were used as experimental populations that represent native and landrace ranges. For the invasive range, we used seeds from maternal plants collected in the field. We germinated and grew one seed per maternal plant; for each population, we randomly paired offspring from different maternal plants for crosses and used seeds from the F_1_ generation as full‐sib families, which we used to represent populations from the invasive range. The steps for artificial crosses are summarized in Figure 2. This generation of seed families was collected from April to May 2015.

### 2.4 Greenhouse experiment

To address our questions, we conducted a greenhouse experiment from August 2015 to February 2016, where seeds from F_1_ families were grown in a common environment using a completely randomized design with 14 populations (divided unequally among three ranges) × 5 families/population × 4 replicates/maternal family (*n* = 280 plants). We planted seeds in the UNM Research Greenhouse in 3.78‐L pots containing a 1:1 mix of sand and Metro Mix^®^ (SunGro Horticulture^®^, Canada). Initially, we used one pot per family (70 pots) and planted approximately 30 seeds in each pot. On the first day of planting, we randomized the location of all 70 pots. As seedlings emerged from each family/pot, we randomly selected and transplanted four seedlings to separate pots. Each seedling that germinated was transferred to a new pot before or at the emergence of the first leaf. After all seedlings were transplanted to individual pots, we randomized pots by using PROC PLAN in SAS 9.3 (SAS Institute) and R Studio (R Core Team, 2018). When we found two or more plants from the same population or maternal family were adjacent to each other, we separated them by assigning new locations. We further controlled for spatial variation in the greenhouse by haphazardly rearranging pot locations for all plants twice at the rosette stage, then twice at the bolting/fruiting stages. We maintained the greenhouse temperature at a minimum temperature of 26.5°C and kept the room at 40% humidity. We supplemented natural lighting with two 1,000 w sodium halide bulbs, so that the photoperiod is constantly at 14 hr days and 10 hr nights.

We hand‐watered all pots until the fourth week after planting, and then used an automated drip system twice per day for four‐minute periods in the morning and in the late afternoon. As the plants grew larger, we incrementally increased watering time per day. We administered 25 ml of 1 g/L Peters^®^ 20–20–20 General Purpose Fertilizer (The Scotts Company) once per week until 95% of the plants reached the flowering stage. To ensure that measurements during the adult stage were not recorded when plants were root‐bound in their pots, most adult trait measurements, except for aboveground biomass, leaf mass, and leaf margin traits, were collected between the time of first bud appearance and 30 days after first budding. When an individual plant reached 30 days after budding, we collected the entire aboveground structure for biomass measurement.

### 2.5 Trait measurements

Over the lifespan of the plant, we measured a total of 33 traits (Table 2). We recognize that some of these traits are correlated with each other and some of these traits may have been affected by pot constraint at some point in the experiment. We corrected for those problems in the following ways. First, we divided variables into five groups, phenological characters, leaf characters, branch architecture, plant size, and reproductive characters. We used principal component analysis to generate one or two composite characters for each of these trait groups. Second, while annual *Brassicas* can be root‐bound in pots and that pot constraint might confound analysis, we did not simply measure traits at the end of the experiment. We reduced the possibility of systematic error by measuring several traits repeatedly during the growth of the plants and all the measures were combined into the appropriate principal components. We surmised that the PCA identifies via loadings the most variable traits. Traits that had stopped changing due to pot constraint would have been less variable and received low loadings in the composite variables.

**Table 2:** Trait groups with their life‐history characters and principal components loadings

Composite trait group	Reasons for trait selection	Individual traits	PC1 loading	PC2 loading
Phenology	Early and rapid phenology confers advantage in desert invasive populations (Marushia et al., 2012, 2010). Phenology determines sowing and harvest time, and yield in *Brassica* crops (Kirkegaard et al., 2016; Wang, Wang, Wang, & Tang, 2012)	Days to appearance of first bud	−0.7	−0.01
		Days to appearance of first flower	**−0.69**	−0.06
		Senescent leaf: young leaf	0.08	0.78
		Days from first bud to first flower	0.17	−0.63
Leaf traits	Leaf traits are associated with fitness in desert annuals (Angert, Horst, Huxman, & Venable, 2010). Leaf size and leaf margin traits are associated with leaf thermoregulation, especially in hot desert habitats (reviewed in Nicotra et al., 2011 and Wright et al., 2017). Leaf size in seed crops, including *Brassica*, is correlated with yield (e.g., Mendham & Scott, 1975).	Leaf length mean—6 days from first bud	−0.46	0.03
		Leaf length mean—12 days from first bud	−0.41	0.05
		Leaf mass per area	−0.14	**0.36**
		Number of indentations	−0.14	0.60
		Number of lobes	−0.05	0.40
		Indentation depth	−0.04	**0.55**
		Indentation width	0.25	0.17
		Lobe width	0.34	0.05
		Leaf width	**0.44**	0.12
		Leaf length mean—30 days from first bud	**0.46**	0.03
Branch architecture	The number of branches, length of branches, and branch angle contributes to shape of *Brassica* (Cai et al., 2016), which can allow a whole *B. tournefortii* plant to disperse seeds by moving as a tumbleweed (B. Alfaro, personal observation). These traits were identified to affect plant movement (whole plant dispersal) in other tumbleweeds in western United States (Baker, 2007; Borger, Walsh, Scott, & Powles, 2007). The number of branches determines yield in *Brassica* seed crop species, branch length associated with inflorescence length in *Brassica* species (Cai et al., 2016).	Number of branches—6 days from first bud	**0.55**	0.24
		Number of branches—18 days from first bud	**0.53**	0.10
		Number of branches—12 days from first bud	**0.46**	−0.07
		Branch length mean—12 days from first bud	0.40	−0.33
		Secondary branch thickness—12 days from first bud	0.19	**−0.43**
		Secondary branch thickness—18 days from first bud	0.10	0.25
		Mean primary branch angle	−0.03	**−0.45**
		Branch length mean—18 days from first bud	−0.03	−0.60
Size	Increased size is associated with invasiveness in invasive plant species (Willis, Memmott, & Forrester, 2002). The size of a *Brassica* plant can be used to determine yield in crop species (Cai et al., 2016).	Height—30 days from first bud	**−0.56**	−0.09
		Height—18 days at first bud	**−0.52**	−0.21
		Aboveground dry biomass	**−0.49**	0.03
		Total number of leaves	−0.26	0.23
		Height—12 days at first bud	−0.26	−0.27
		Height—6 days at first bud	0.11	−0.65
		Height at first bud	0.13	−0.64
Reproduction	Related to fitness traits; can be considered as a fitness component; associated with propagule pressure and yield	Total flower count—12 days from first bud	**0.59**	−0.38
		Total bud count—12 days from first bud	**0.58**	−0.41
		Total bud count—6 days from first bud	0.44	0.53
		Total flower count—6 days from first bud	0.36	0.64

Note

The most strongly loaded trait for each principal component axis used for analyses is indicated in bold.

#### 2.5.1 Phenology

In the southwestern United States, *B. tournefortii* can outcompete native plants by emerging earlier and reproducing rapidly than the native plants (Marushia et al., 2012, 2010). We have observed that, in areas that do not experience snow or frost in early spring or late winter, some populations can produce seeds at the onset of the growing season, allowing them to avoid possible mortality from aridity late in the growing season (B. Alfaro personal observation). But while rapid reproductive phenology is critical for this plant to succeed in its North American range, the crop fields of landrace populations may have led to slower reproductive phenology. In *Brassica* used for canola, increasing day length and warmer temperature is important for development of inflorescences (Burton et al., 2008). Most importantly, the length of the reproductive period is an evolutionary response of many desert annuals to cope with aridity (Kemp, 1983). To quantify reproductive phenology, we recorded the date of appearance of the first bud and the first flower. We calculated the days from bud to flower, which marks the days between the appearance of the first bud to the appearance of first petals. To measure senescence for each plant, we counted the number of senesced leaves 30 days after the appearance of the first bud.

#### 2.5.2 Leaf traits

The common limiting factor in all our source populations is aridity. To cope with variability in amount of moisture in hot environments, different species of desert annuals have modified the sizes and shapes of their leaves to increase water use efficiency and reduce leaf damage (reviewed in Wright et al., 2017). Therefore, we included a panel of leaf morphological traits related to leaf size and shape that are critical for plant survival in desert habitats. We measured length of two tagged leaves for each plant at 6, 12, and 18 days from first bud. For leaf mass and leaf margin traits, we collected the tagged leaves at 30 days after budding. We measured leaf margin architecture to assess the potential for adaptation. Specifically, number of lobes per leaf, lobe width, leaf width, number of indentations (teeth) per leaf per plant, distance between indentations, as well as indentation depth per leaf were measured. These characters may be related to leaf function of different temperatures. We used the program LAMINA (Bylesjo et al., 2008) to obtain these leaf measurements. We collected two fresh leaves for each plant, and then obtained digital images by scanning them at 200 dpi using a Hewlett‐Packard CP1210 scanner or taking digital photographs of leaves using either an iPhone 6S (Apple Inc.) or Samsung Galaxy Note8 (Samsung Group) clamped at 0.25 m height on a metal stand with ambient lighting on a white, nonreflective surface. Using a reference image, we analyzed all digitized leaf images using the LAMINA software.

In addition to leaf margin structure, we also measured leaf mass per area. To determine leaf mass per area, we collected two leaves per plant, pressed them for 24 hr, scanned them at 200 dpi using a Hewlett‐Packard CP1210 scanner, and then used LAMINA to measure area of each leaf. We did not keep track of leaf phenology per plant, so to make sure that we sampled leaves at a consistent phenological age at the time of collection, we chose the largest leaves. Next, we dried the leaves in a desiccator oven at 45°C for 7 days before weighing them on a Mettler Toledo AG135 analytical balance (Columbus, OH) to the nearest 0.0001 g. To measure lobe width for each dried leaf, we first located the lobes at the midpoint from the base to the tip of each sampled leaf. We then used a digital caliper to measure the width of the right and left mid‐lobes to the nearest 0.01 mm. We used the mean leaf lobe width of two leaves per plant for our analysis.

#### 2.5.3 Branch architecture

Adaptations to disperse seeds for population and range expansion is critical for plant survival and establishment in desert environments (Fllner & Shmida, 1981). Invasive *B. tournefortii* in North America is known to disperse fruits and seeds in the southwestern United States by moving as a tumbleweed (Buckley, 1981), but traits associated with this dispersal mode have not been shown as adaptive in this species. We chose branch architecture traits based on Baker, Beck, Bienkiewicz, and Bjostad (2008) finding that variation in branch density and morphology in different populations of tumbleweed species (*Centaurea diffusa*, *Kochia scoparia*, and *Salsola* spp.) was associated with each population's proportion of mobile plants.

We tagged two terminal branches situated at mid‐height of the plant for branch measurements. We measured branch length with a meter stick to the nearest 0.1 cm and branch thickness using a digital caliper to the nearest 0.01 mm at 12 and 18 days after the appearance of the first flower bud. If the tagged branch was bent due to the weight of fruits, we straightened it before measurement. To determine total branch number, we counted the total number of terminal (secondary) branches per plant at 6, 12, and 18 days from first bud. In addition to branch length and number of branches, we measured thickness at the base the branch and branch angle. Thirty days after budding of each plant, when the plants were fully grown, we haphazardly selected two primary branches and measured their angles with respect to the main branch using a protractor to have a rudimentary measurement of branching pattern and plant shape.

#### 2.5.4 Plant size

We included plant size as a trait group because it is known in crop *Brassica* (e.g., Mendham & Scott, 1975) that the size of the plant can associate with reproductive output and therefore affect fitness. We measured shoot height for each plant at the appearance of the first bud and 6, 12, 18, and 30 days from first bud. At 30 days after appearance of the first bud, we counted the total number of basal and cauline leaves per plant. To measure aboveground biomass, each plant was excised from the roots at 30 days after appearance of the first bud. The samples were then placed in a paper bag, cut into smaller sections, stored at room temperature (~25°C) for 1 month or longer, and then dried for 48 hr in a desiccator oven at 65°C before weighing. Mass of leaves removed to calculate leaf mass/area was added to these measurements.

#### 2.5.5 Reproduction

For native, invasive, and landrace populations, the number of reproductive parts in *B. tournefortii* produced can determine the survival success, propagule pressure, or yield of a population (e.g., Trader et al., 2006). In preliminary analyses from pilot studies, we have determined that the amount of buds or flowers are associated with the number of fruits produced by a plant, which is associated with seed production in this species. To measure variation in reproductive traits, we counted the total number of flower buds and total number of flowers at 6 and 12 days after the emergence of the first bud for each plant.

#### 2.5.6 Relative fitness

We chose total number of fruits at 18 days after budding as a fitness component and as a proxy for relative fitness. We understand that fruit production does not entirely represent relative fitness. However, fruit production has been shown to contribute to successful establishment in this species (Abella et al., 2013; Bangle et al., 2008; Gorecki et al., 2012; Trader et al., 2006). In landrace populations, increased number of fruits is commonly selected by breeders, especially for seed crops (Tester & Langridge, 2010). And, in invasive populations in the southwestern United States, it has been hypothesized that high fruit number results in increased propagule pressure (Bangle et al., 2008; Trader et al., 2006). Although we have repeated measurements of fruit number, measurements earlier than 18 days after budding do not represent total fruiting output because flowers and buds are still present. Measurements at 18 days or later after budding, on the other hand, are taken when all viable flowers have produced fruits. In addition to being fully set with fruit and less pot constrained compared to plants at 30 days post budding, we chose total fruit number per plant at 18 days after budding because of its correlation with other traits that we identified in a pilot study. We determined relative fitness of each plant by identifying the sample plant with the most fruits for our entire study, then calculating the relative fitness of each plant as fruit number of a plant/fruit number of the plant with the most fruits.

### 2.6 Statistical analysis

We narrowed the number of traits to analyze by using PCA via data matrix. First, we divided traits into five groups: phenology, leaf morphology, branch architecture, size, and reproduction. Using *prcomp* in R Studio (R Core Team, 2018), we ran separate PCA procedures for each trait group, and then used the factor scores for the first or first and second principal component for each group as new variables to have a manageable number of variables for the remaining analyses. The first principal components explained the following proportions of the variance in their trait groups: phenology PC1, 50.9%; leaf PC1, 30.7%; branch PC1, 24.9%; size PC1, 32.3%; and reproduction PC1, 46.5%. For phenology PC1, days to appearance of first bud (−0.70) and days to appearance of first flower (−0.69) were most heavily loaded and both negatively correlated relative with all phenology variables (Table 2). We included second principal components for leaf and branch traits, which explained the following proportions of variance in their trait groups: leaf PC2, 22.0% and branch PC2, 15.0%. For the leaf PC1, mean leaf length at 30d from first budding (0.46) had the highest loading along with leaf width (0.44, Table 2). For the leaf PC2, the number of indentations per leaf (0.60) and indentation depth (0.55) had the highest loadings. Because leaf PC2 differed among imaging devices, we obtained the residuals from a one‐way ANOVA (leaf PC2 = device) and used the residual values for all our analyses. Among all traits in branch PC1, the number of branches 6 days after appearance of first bud (0.55) has the highest loading. For branch PC2, branch length at 18 days after appearance of the first bud (−0.60) and branch angle (−0.45) had the highest loadings and are negatively correlated to all branch architecture variables. For size PC1, the variable with the highest loading was plant height at 30 days after first budding (−0.56), which is negatively correlated with all other size variables. For the reproduction PC1, the variable with the highest loading (0.59) was total flower number at 12 after first bud.

We performed mixed‐model ANOVAs in SAS 9.4 (SAS Institute) with trait group principal components as dependent variables. The independent variables were range (native, invasive, and landrace) as a fixed effect, experimental population nested within range as a fixed effect, and maternal family within population as a random effect. We analyzed relationships of phenology, leaf morphology, branch architecture, size, and reproduction principal components with relative fitness using two statistical approaches. We knew from preliminary analysis that some traits have nonlinear fitness functions; therefore, our first step was to plot these fitness functions in each range. To avoid forcing regression lines into either linear or quadratic fits and to capture nonlinear trends, we used a general additive model (*gam*) approach to smooth regression lines. In particular, we used the *gam* function in the *mgcv* (Wood, 2000) and *ggplot2* (Wickham, 2016) packages in R. Second, we performed separate type III ANCOVAs via *glm* in R Studio for each trait suite. Based on our *gam* regression lines and preliminary model selection procedures in a pilot study, we used a general model form to test directional and nonlinear selection for all trait suites: relative fitness (*w*) was the response variable, range was the categorical variable, and the linear (*β*) and quadratic (*γ*) terms for all composite traits were covariates. We also included the interaction of the covariates with range in our models. While *gam* results test smoothing parameters for predictor variables, they do not include parameter estimates that are relevant for interpreting phenotypic selection. So, we used the ANCOVA results to interpret the regression lines of fitness functions; that is, we used the sign and value of estimates of regression coefficients to indicate the type, direction, and magnitude of phenotypic selection. Specifically, a significant *β* is interpreted as directional selection, a significant negative curve (−*γ*) is interpreted as stabilizing selection, and a significant positive (+*γ*) is associated with disruptive selection (Conner & Hartl, 2004; Lande, 1991).

We used aridity index to determine how desert climate can affect population means and selection strength (Trabucco & Zomer, 2019). While climate variables such as BioClim can be used for our analysis (Hijmans, Cameron, Parra, Jones, & Jarvis, 2005), aridity index is derived from both temperature and moisture, as well as potential evapotranspiration. For desert habitats, this can be more biologically meaningful in terms of fitness of plants. After obtaining the aridity index for all sites, we ran ANCOVA tests using the model form trait group PC = aridity index + range + aridity × range, to test presence and/or changes in clinal trends to detect possible signals of rapid evolution. We used the *ggplot2* package in R Studio to plot models to graphically assess potential clinal trends.

Lastly, we asked whether the strength and direction of selection changed among populations along environmental gradients. We used the same approach proposed by Wade and Kalisz (1990), in that we regressed a climate variable (aridity index) versus linear and quadratic population selection gradients. However, instead of examining variation in selection strength in a habitat, as performed by Stewart and Schoen (1987), we extended this approach to the scale of range‐wide climatic gradients. To determine differences in selection intensity along climate gradients, we split the dataset by populations, so we could calculate both linear and quadratic slope estimates for each individual population. We were generally interested in how magnitude and direction change across environments, so we did not obtain absolute values of the selection gradients. We then performed ANCOVA (type III SS) to test if slopes of the environment PC versus selection strength (i.e., population selection gradient) for focal trait PCs were different between ranges and used *ggplot2* in R Studio to delineate these patterns.

## 3 RESULTS

### 3.1 Variation of trait PCs and relative fitness

**Figure 4:**
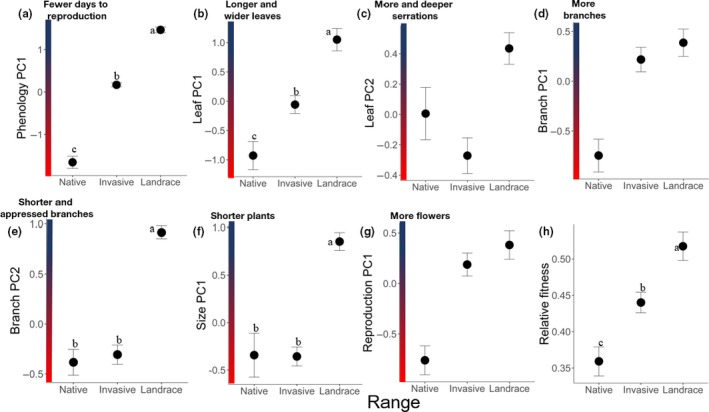
Range means of trait principal components (black circles) in native (*N* = 77), landrace (*N* = 72), and invasive (*N* = 117) ranges: (a) phenology PC1 (days to first bud), (b) leaf PC1 (mean leaf length), (c) leaf PC2 residuals (number of indentations per leaf), (d) branch PC1 (number of branches), (e) branch PC2 (lateral branch length), (f) size PC1 (plant height at 30 days after first bud), and (g) reproduction (total number of flowers). The range means of relative fitness are also shown (h). Means within figures that have different superscripts are significantly different in Tukey HSD comparisons

The onset of reproduction was earliest (phenology PC1) in the landrace populations, intermediate in the invasive populations, and latest in the native populations. These differences are statistically significant (Table 3, Figure 4a). Leaf PC1, strongly loaded for leaf size, was largest in the landrace populations and smallest in the native populations. These differences are statistically significant (Table 3, Figure 4b). For leaf PC2, which was highly loaded for number and depth of leaf indentations, the landrace range had the most serrated leaves, but ranges did not differ significantly in indentations (Table 3, Figure 4c). Branch PC1, which was most strongly influenced by number of branches, did not vary significantly among ranges (Table 3, Figure 4d). In contrast, means of branch PC2 (negatively loaded for lateral branch length and angle) were significantly different among ranges with the native and invasive populations having longer, wider‐angled branches than the landraces (Table 3, Figure 4e). While the native range did not differ in mean size PC1 (plant height) from the invasive populations, the landrace ranges had significantly shorter plants than native and invasive ranges (Table 3, Figure 4f). Landrace and invasive ranges produced more flowers (reproduction PC1) compared to the native range; this difference approached significance (Figure 4g). Mean relative fitness was highest in the landrace range, intermediate in the invasive range, and lowest in the native range (Figure 4h). These differences were statistically significant (Table 2).

**Table 3:** Mixed effects ANOVA results for principal components of phenology, leaf, branch architecture, size, and reproduction traits

Trait group	Source	*Df*	*F^p^*	*R* ^2^
Phenology PC1 (number of days to first bud)	Range	2	35.79****	0.90
	Population within range	4	17.75****	
	Maternal family	44	3.14****	
Leaf PC1 (mean leaf length)	Range	2	5.43**	0.58
	Population within range	4	12.46****	
	Maternal family	44	2.27****	
Leaf PC2 residuals (number of leaf indentations)	Range	2	0.28	0.32
	Population within range	4	0.51	
	Maternal family	44	1.15	
Branch PC1 (total number of branches per plant)	Range	2	1.71	0.48
	Population within range	4	5.10***	
	Maternal family	44	1.88**	
Branch PC2 (lateral branch length)	Range	2	3.36*	0.50
	Population within range	4	2.43*	
	Maternal family	44	1.22	
Size PC1 (plant height at 30 days after first bud)	Range	2	3.47*	0.66
	Population within range	4	34.94****	
	Maternal family	44	1.61*	
Reproduction PC1 (total numbers of flowers per plant)	Range	2	2.58†	0.43
	Population within range	4	4.13**	
	Maternal family	44	1.97***	
Relative fitness, *w*	Range	2	6.13**	0.42
	Population within range	4	0.73	
	Maternal family	44	1.88**	

Note

Relative fitness was also included (*n* = 266).

^†^ *p* ≤ .1.

* *p* ≤ .05.

** *p* ≤ .01.

*** *p* ≤ .001.

**** *p* ≤ .0001.

### 3.2 Between range differences in fitness functions

**Figure 5:**
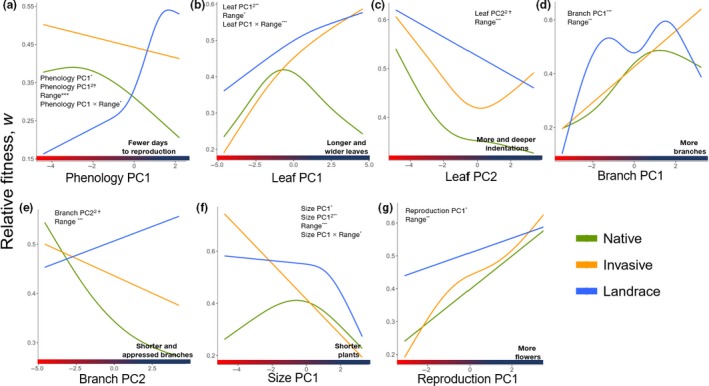
Plots of fitness functions for (a) phenology PC1 (days to first bud), (b) leaf PC1 (mean leaf length), (c) leaf PC2 residuals (number of indentations per leaf), (d) branch PC1 (number of branches), (e) branch PC2 (lateral branch length), (f) size PC1 (height), and (g) reproduction PC1 (total number of flowers) in native (*N* = 77), landrace (*N* = 72), and invasive (*N* = 117) ranges. The *x*‐axes are values of composite trait groups (PCA scores), and the *y*‐axes are relative fitness (*w*) values derived from maximum total number of fruits per plant. To detect unknown nonlinear trends, generalized additive model (gam) function for regression line smoothing (*k* = 5 dimensions) was used within the *ggplot2* package in R Studio. Full model descriptions are in Table 4. *p* ≤ .1^†^, *p* ≤ .05*, *p* ≤ .01**, *p* ≤ .001***, *p* ≤ .0001****

In the following analyses, we were interested in effects of the trait PC values on fitness (a significant trait effect is an overall linear effect and a significant trait^2^ effect is an overall quadratic effect) and whether these fitness functions differed among ranges. A significant trait‐by‐range effect indicates that the slope of the fitness function differed among ranges and a significant trait^2^‐by‐range effect indicates that the shape of the fitness function differed among ranges. While there were a number of trait, trait^2^, and trait‐by‐range effects, we did not observe significant effects of trait^2^‐by‐range on relative fitness (Table 4). This result would suggest that the fitness functions are similar in shape across ranges; however, when we plotted fitness functions for each range, we observed nonlinear trends in most traits (Figure 5). Among the nonlinear regression lines, six are from the native range and four of these plots show evidence of stabilizing selection (Figure 5a–d). The invasive and landrace ranges, on the other hand, show mostly directional selection. However, in the landrace range relative fitness increases with shorter reproductive periods (Figure 5a), and in the invasive range leaf PC2 has maximum fitness in extreme leaf margin phenotypes.

**Table 4:** ANCOVAs of fitness functions among native, invasive, and landrace ranges (*n* = 266)

Composite trait variables	Source	*df*	*F^p^*	*R* ^2^
Phenology PC1 (number of days to first bud)	Trait	1	4.69*	0.12
	Trait^2^	1	3.17†	
	Range	2	7.42***	
	Trait × Range	2	4.30*	
	Trait^2^ × Range	2	0.05	
Leaf PC1 (mean leaf length)	Trait	1	0.70	0.24
	Trait^2^	1	9.27**	
	Range	2	4.01*	
	Trait × Range	2	7.64***	
	Trait^2^ × Range	2	0.53	
Leaf PC2 residuals (number of leaf indentations)	Trait	1	0.0089	0.14
	Trait^2^	1	2.86†	
	Range	2	13.08***	
	Trait × Range	2	0.03	
	Trait^2^ × Range	2	0.79	
Branch PC1 (total number of branches per plant)	Trait	1	15.09***	0.34
	Trait^2^	1	2.41	
	Range	2	7.02**	
	Trait × Range	2	0.95	
	Trait^2^ × Range	2	0.91	
Branch PC2 (lateral branch length)	Trait	1	1.90	0.15
	Trait^2^	1	2.81†	
	Range	2	8.30****	
	Trait × Range	2	0.99	
	Trait^2^ × Range	2	2.14	
Size PC1 (plant height at 30 days after first bud)	Trait	1	4.49*	0.25
	Trait^2^	1	10.58**	
	Range	2	10.71***	
	Trait × Range	2	4.00*	
	Trait^2^ × Range	2	1.80	
Reproduction PC1 (total numbers of flowers per plant)	Trait	1	6.32*	0.19
	Trait^2^	1	0.40	
	Range	2	5.31**	
	Trait × Range	2	0.99	
	Trait^2^ × Range	2	0.10	

Note

The independent variables are range, linear, and quadratic terms for composite trait variables (covariates), and the interactions of range with the trait covariates. The dependent variable is relative fitness, calculated as sample number of fruits/maximum number of fruits. Adjusted *R* ^2^ values are included.

^†^ *p* ≤ .1.

* *p* ≤ .05.

** *p* ≤ .01.

*** *p* ≤ .001.

**** *p* ≤ .0001.

### 3.3 Clinal patterns of population means and strength of selection

**Figure 6:**
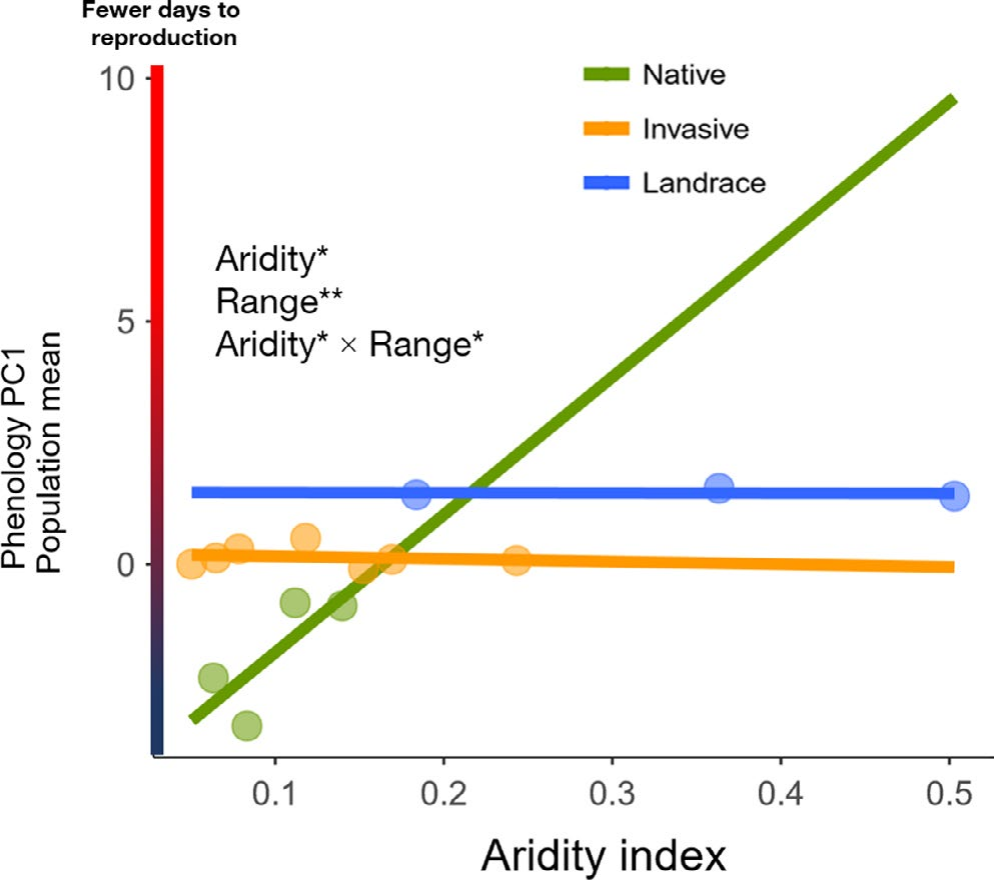
Regression lines of aridity index versus population means of phenology PC1 (*n* = 14). Significant main and/or interaction effects from ANCOVA tests are shown (*p* ≤ .1^†^, *p* ≤ .05*, *p* ≤ .01**, *p* ≤ .001***, *p* ≤ .0001****)

**Figure 7:**
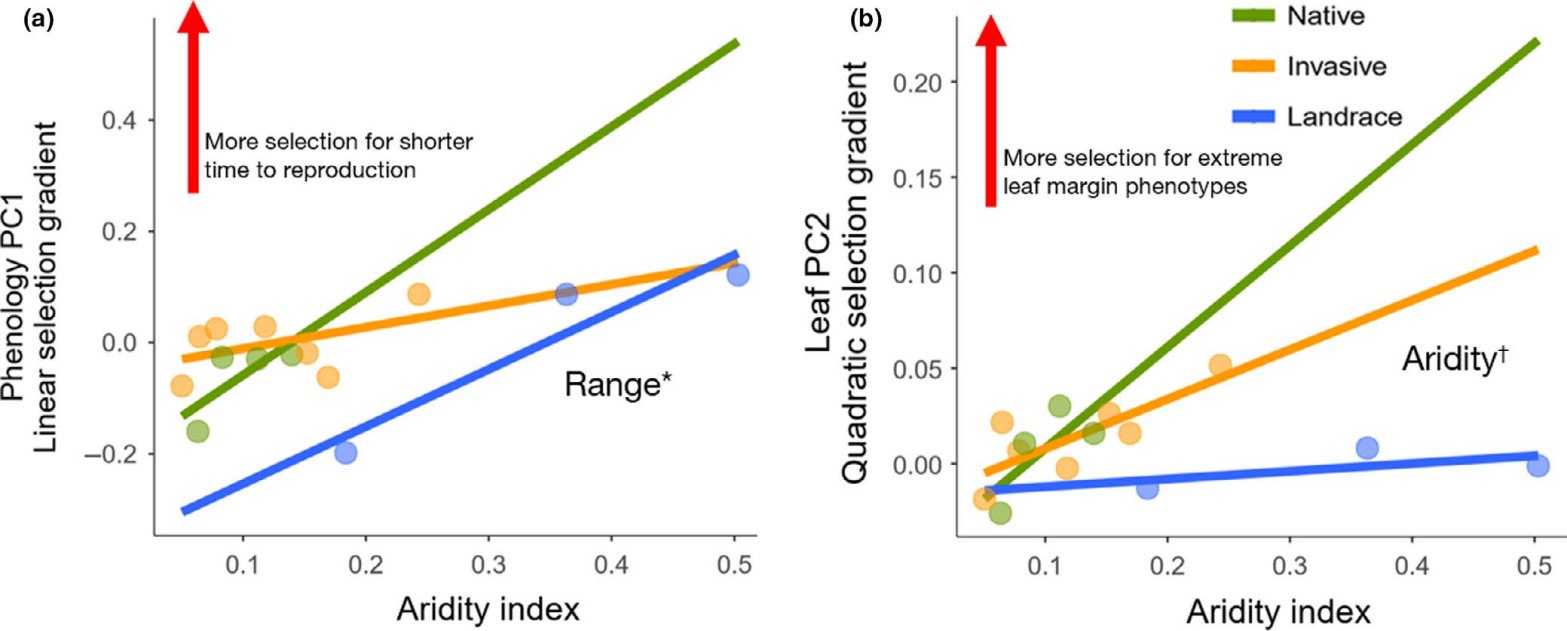
Regression lines of aridity index versus population selection gradients of phenology PC1 and leaf PC2 in the native, invasive, and landrace ranges (*n* = 14). Significant main and/or interaction effects from ANCOVA tests are shown (*p* ≤ .1^†^, *p* ≤ .05*, *p* ≤ .01**, *p* ≤ .001***, *p* ≤ .0001****)

Of all composite traits that showed genetic bases for variation and relationship with fitness, phenology PC1 (reproductive phenology) and leaf PC2 (highly loaded for leaf indentation depth and leaf mass per area) also had statistically significant relationships with aridity index when clinal trends of the two traits were analyzed at the population level. When phenology PC1 in populations was compared along aridity gradients among the three ranges, the invasive and landrace populations showed no change in timing of reproduction, as indicated by flat trendlines (Figure 6). In contrast, the three native populations had shorter times to reproduction with lower aridity, showing a steep increasing cline (Figure 6). The strength of directional selection, measured as the linear slope of trait value versus relative fitness in each population, varied for phenology. Specifically, selection for shorter reproductive periods increased with increasing humidity for the native, invasive, and landrace populations (Figure 7a), but patterns did not statistically vary among ranges, even though the native and landrace populations had steeper trends than the invasive populations.

While phenotypic means of leaf PC2 (highly loaded for leaf indentation depth and leaf mass per area) did not have statistically important associations to aridity index, nonlinear selection in populations changed with increasing humidity in some ranges, as indicated by increasing lines (Figure 7b). Both native and invasive populations showed stronger selection for extreme phenotypes with increasing humidity. Landrace populations had an almost flat trendline with an intercept below zero, indicating that nonlinear selection in these populations is weak regardless of the amount of aridity.

## 4 DISCUSSION

Associations of phenotypic variation with environmental conditions are commonly observed in plant populations. But, different variability of climate can alter timing of environmental cues that dictate resource availability for plants (i.e., water). Sometimes, this can stimulate evolution of new patterns of phenotypic differentiation and selection (Franks, Sim, & Weis, 2007; Nicotra et al., 2010), as we have observed in our comparison of phenotypic means and fitness functions between native, invasive, and landrace populations of *Brassica tournefortii*. While the type of among‐range phenotypic differentiation seen in our study system has been attributed to rapid evolution in invasive and landrace plants in other species (Buswell et al., 2011; Colautti & Lau, 2015) the reasons why a certain feature will have higher or lower phenotypic means at a certain region are complex. Comparing range means is suggestive, but not conclusive. By including fitness functions in our analyses, we were able to test whether the variability and differentiation in traits among ranges are likely to be associated with fitness. While we expected some fitness functions to differ among ranges, we did not have specific predictions for each trait for each range. The native fitness functions, which showed nonlinear patterns, fit Endler's (1986) prediction that phenotypic variation in demes that underwent extended periods of local adaptation will stabilize to intermediate phenotypes, except perhaps reproductive traits. In contrast, invasive and landrace ranges had fitness functions that are mostly directional, which we interpret as indicating rapid evolution.

Our pooled analyses allowed us to describe a snapshot of phenotypic evolutionary potential in entire ranges in terms of composite trait means and fitness functions. We were also able to identify that phenotypic means and selection strength of composites of leaf margin and phenology traits can vary across each range as a response to a critical limiting factor, aridity, in our study area. While the three ranges are all hot environments, they vary in vegetation types, topography, and aridity (Laity, 2008). Further, the contemporary evolutionary histories are different for the native, invasive, and landrace populations we included in our study. Based on our findings, we assert that the native populations in Israel, Morocco, and Spain have adapted to Mediterranean ecosystems, possibly through millennia, while the younger populations in the southwestern United States have been recently established in mostly roadsides and washes. It is worth noting that the invasive populations we studied are experiencing frequent boom‐and‐bust cycles due to the highly variable precipitation in this region, which can contribute to genetic differentiation (Li et al., 2015). The clinal patterns we determined indicate that aridity is a likely agent of selection for *B. tournefortii*, which means it may have affected genetic differentiation among populations and among ranges. Thus, we expected to find patterns suggesting adaptive or maladaptive phenotypic differentiation for ecologically important traits, as Winkler, Gremer, Chapin, Kao, & Huxman, 2018 have identified in other populations of *B. tournefortii*. This was true for one composite trait, phenology PC1, which had a defined cline for the native populations, but relatively neutral or flat clines for invasive and landrace ranges. While the neutral patterns for the invasive and landrace ranges do not indicate genetic or phenotypic differentiation, mechanisms such as phenotypic plasticity can produce consistent phenotypes, such as in reproductive phenology (Richards, Bossdorf, Muth, Gurevitch, & Pigliucci, 2006).

We found trait means and fitness functions that varied among ranges, but these traits did not show any clinal signal that aridity was critical to their survival. While including other climate features in our study may seem to be a prudent approach, we were not confident that the number and locations of source populations in our study represented the full spectrum of variability required for a three‐way analysis. We acknowledge that this study would have stronger implications if the number of populations had been balanced among ranges. We are also aware that the accessions we used were collected in different years, which could have confounded our estimates of selection strength even though seeds used for our study were produced in a common greenhouse. Nonetheless, our common greenhouse experiment shows that even with limited numbers of populations, significant shifts in clinal patterns of trait means and selection gradients between ranges can be detected.

If the presence of a cline between a trait mean and an environmental variable is considered a signal of local adaptation, then differences between native, invasive, and landrace clines indicate rapid adaptation to novel environments (Colautti & Barrett, 2013; Colautti & Lau, 2015). If we examined just regression lines of aridity versus composite trait means, then we would have concluded that invasive and landrace populations both have weak or no signal for local adaptation for reproductive phenology and leaf margin morphology, with respect to aridity index. However, patterns of selection strength across native, invasive, and landrace aridity gradients tell a different story. We highlight phenology for the rest of our discussion, as it showed signals of adaptive variation among range means, fitness functions, and among clines of population means and population selection gradients.

In some cases, episodes of rapid adaptation occur due to changes in genetic composition driven by a combination of long‐distance dispersal events and altered gene flow (Colautti & Lau, 2015; Dlugosch & Hays, 2008). In *B. tournefortii*, possible bottleneck effects in invasive populations and the intentional selection of maternal phenotypes in the landrace populations may have led to neutral patterns for mean phenology (Figure 6). Neutral patterns of mean time to reproduction in the invasive and landrace ranges suggest a type of plasticity in which different genotypes express the same phenotype in different environments (Richards et al., 2006). In the invasive range, where climate varies dramatically, consistent phenology gives an edge against endemic plants if *B. tournefortii* can reproduce consistently earlier (Marushia et al., 2012).

Traditional agricultural practices in the landrace range of *B. tournefortii* appear to have led to consistent reproductive phenology even with highly variable aridity. That is, the three landrace accessions we studied showed stability. The clines we delineated for landraces suggest that growers may have artificially selected for the most productive plants with the shortest growth periods, which can allow efficient and consistent harvest. As a result, a shorter mean growth period before reproduction may have evolved in landrace *B. tournefortii* allowing plants to rapidly allocate resources to seeds with limited water.

In competition experiments, invasive *B. tournefortii* outcompeted other non‐native *Brassicaceae* with its rapid seedling and reproductive phenology (Marushia et al., 2012, 2010). Based on our findings, invasive mustard is rapidly evolving faster mean growth periods until reproduction, but the trend is not as strong as native and landrace ranges (Figure 7a). Perhaps plants with the fastest phenotypes are the ones that can form monocultures that fill vacant niches in the southwestern deserts of North America (Li et al., 2015). With potentially high intraspecific competition, however, there is the possibility of a fitness cost from a correlated trait that drives negative selection for rapid growth and reproduction (Bossdorf, Prati, Auge, & Schmid, 2004).

## 5 CONCLUSIONS

The ability to establish in extreme arid habitats makes *B. tournefortii* formidable to control because of diverse niches it can occupy. Although the results are complex, some traits have rapidly diverged among ranges and among populations. Rapid adaptation of phenology to varying degrees of aridity may have resulted in plants that are more suited to their new environments, which is a plausible hypothesis for the spread of *B. tournefortii* in the southwestern United States in less than a century. On the other hand, breeding programs for *Brassica* seed crops should aim to achieve stable phenology to have plants that can withstand the rapid changes in local and global climates.

## CONFLICT OF INTEREST

The authors do not declare any conflict of interest.

## AUTHOR CONTRIBUTIONS

B. A. formulated the questions, conducted the experiment, analyzed the data, and wrote the manuscript. D. L. M. provided mentorship throughout the entire study, especially with analysis, and participated substantially in writing the manuscript.

## Data Availability

All figures in the article can be accessed via FigShare at https://doi.org/10.6084/m9.figshare.9916178.
